# “Eco-friendly HPLC method for analysis of dipyrone and hyoscine in different matrices with biomonitoring”

**DOI:** 10.1038/s41598-024-71046-6

**Published:** 2024-09-18

**Authors:** Reem A. El kalla, Nermine S. Ghoniem, Hala E. Zaazaa, Ahmed Emad El Gendy, Ghada A. Sedik

**Affiliations:** 1 Analytical Chemistry Department, Faculty of Pharmacy, Misr International University, Km 28 Misr-Ismailia Road, Cairo, Egypt; 2 Pharmaceutical Analytical Chemistry Department, Faculty of Pharmacy, Kasr El Aini, Cairo University, Cairo, 11562 Egypt

**Keywords:** Dipyrone, Hyoscine, 4- aminoantipyrine, Tropic acid, HPLC bioanalytical studies, Green chemistry, Analytical chemistry, Green chemistry

## Abstract

A selective, precise, and accurate reversed HPLC method has been developed and validated for simultaneous separation and determination of two veterinary drugs, dipyrone and hyoscine, in their combined dosage form in the presence of their official impurities, namely 4-aminoantipyrine and tropic acid, in addition to the formulated preservative: phenol. The linearity range was found to be (1.00–35.00 µg/mL) for dipyrone and (2.50–50.00 µg/mL) for hyoscine. It exhibited a satisfactory linearity regression R (0.9999) for both drugs with LOD 0.22 µg/mL and 0.72 µg/mL and LOQ 0.65 µg/mL and 2.19 µg/mL for dipyrone and hyoscine, respectively. Additionally, the two cited drugs were also determined in the presence of dipyrone active metabolite 4-aminoantipyrine using diclofenac as an internal standard in bovine urine. The linearity range was found to be (15–75 µg/mL) for dipyrone, (2.5–60 µg/mL) for hyoscine, and (2.5–60 µg/mL) for 4-aminoantipyrine with linearity regression R (0.9999–0.9998). The LLOQ (15, 2.5, 2.5 µg/mL), LQC (45, 7.5, 7.5 µg/mL), MQC (55, 25, 25 µg/mL), and HQC (60, 50 50 µg/mL) were determined for dipyrone, hyoscine and 4-aminoantipyrine, respectively. UV detection was carried out at 220 nm. The method was validated according to the ICH guidelines, as well as according to FDA guidelines for determining both drugs in bioanalytical matrices and both proved accuracy and precision. A statistical comparison was made between the results obtained and those obtained by the reported method, showing no significant difference in accuracy and precision at p = 0.05. The suggested method was proved eco-friendly through a greenness assessment using two different tools (The analytical eco-scale scored 83, and the AGREE-Analytical Greenness Metric approach scored 0.83). The suggested method can be used in the routine work of quality control labs, screening for drug abuse, and ensuring clean sport for horse racing.

## Introduction

Veterinary drugs are mainly concerned with preventing, controlling, and treating some diseases affecting animals, thus preventing their transmission to people. When a veterinary medicine is prescribed, it must not only be effective but must also be safe for both animals and humans^1^. Detection of veterinary drugs and their residues ensures human health protection through supervision and control^2^.

Impurity profiling and stability studies are crucial to make sure that the drug maintains its high quality and pharmacological effect after leaving the production line until the expiry date^3^. The presence of an impurity in a pharmaceutical dosage form may cause adverse effects for the animal and consumer, alters the dissolution and solubility of the drug constituent, and may affect systemic circulation; it also can threaten animal safety in addition to affecting the biopharmaceutical behavior of the drug substance^4^.

Detection and analysis of veterinary drugs and their metabolites in the urine of live animals are widely used. They are very effective as they have the advantage of retesting animals in case of a suspect result. Furthermore, the concentration of drug residues in urine tends to be higher than in ^5^.

Dipyrone (DIP), Fig. S1, also known as metamizole chemically known as [(1,5-dimethyl-3-oxo-2-phenylpyrazol-4-yl)-methylamino]methanesulfonic acid^6^ is a popular analgesic, non-opioid drug commonly used in human and veterinary medicine as an antipyretic and spasmolytic agent^7^. Hyoscine (HBB), Fig. S1, is chemically known as [(1*S*,2*S*,4*R*,5*R*)-9-butyl-9-methyl-3-oxa-9-azoniatricyclo[3.3.1.0^2,4^]nonan-7-yl](2*S*)-3-hydroxy-2-phenylpropanoate;bromide^6^.

DIP and HBB combined dosage form has an analgesic and spasmolytic effect, which is used in veterinary medicine as an intravenous or intramuscular injection for the treatment of abdominal pain associated with cramps in cattle and horses^8^.

After parenteral administration of DIP, about 90% of the administered dose is excreted in urine as four main metabolites among them is 4- aminoantipyrine (AMP)^9–12^ (Fig. S1) which is chemically known as 4-amino-1,5-dimethyl-2-phenylpyrazol-3-one^13^. It is considered as impurity B of DIP^14^. It is less active than the parent drug, DIP^9,15^. Its presence lessens the activity of DIP despite having a role as an anti-inflammatory, analgesic, and antipyretic drug^16^.

HBB can cause anticholinergic intoxication due to its misuse. Being an easily obtained over-the-counter drug, if it is abused, it can cause hallucinations, the desired effect for abusers. Severe complications are usually associated with anticholinergic and psychosis symptoms. The abuse of this drug can be detected in urine by analysts and forensics in case of death^17^. The majority of the dose is excreted unmetabolized if administered parentally^18,19^.

Tropic acid (TRO), Fig. S1, chemically known as 3-hydroxy-2-phenylpropanoic acid, is considered impurity B of HBB^14^. It has a role as a human xenobiotic functionally related to propionic acid and hydrotropic acid, but its presence indicates the impurity or degradation of HBB^20^ which will decrease the effectiveness of the formulation. Phenol (PHEN) is used as a preservative. Being a phenolic derivative makes it effectively active against gram-positive and negative bacteria^21,22^.

Administration of drugs to enhance the performance of a horse in a sport or race (doping) might be due to the use of a drug intended to treat a specific condition, and there are certain prohibited drugs^23^.According to the FEI (Federation Equestrian International), both DIP (NSAID) and HBB (acts on the parasympathetic) are considered doping drugs for racehorses, and they are prohibited to allow clean horse racing^24,25^, thus, it is of great importance to screen the winning horses’ urine to make sure it is free from these drugs to allow clean sport.

Green chemistry has become essential to avoid hazardous chemicals and methodologies nowadays. Furthermore, green chemistry helps to meet both environmental and economic goals by focusing on the use of safer chemicals, safe elimination of wastes and reagents, lower energy consumption, and real-time analysis^26^.

After surveying the literature, some methods for the simultaneous determination of HBB and DIP, either separately or in combination with other drugs, were found, including spectrophotometric methods^27–31^, electrochemical assays^32–35^, TLC densitometric techniques^36,37^ and additionally, HPLC methods^38–42^.

No method determines the cited drugs in the presence of their official impurities or real urine samples. Thus, our primary goal was to develop an isocratic, specific, eco-friendly, novel, easy and validated HPLC method for the determination of DIP and HBB in the presence of their related impurities (AMP and TRO) and PHEN as a preservative^21,22^ in their dosage form as their presence decreases the effectiveness of the drug as AMP reported to be less active than DIP^9^. While TRO is considered an impurity of HBB^14^.

The method was further applied for assaying DIP, HBB, and AMP as DIP's active metabolites in spiked bovine urine and real samples, using DIC as an internal standard to allow drug monitoring and control doping for racehorses. The drugs are considered prohibited by FEI as they are anti-colic drugs. Consequently, the method can be used effectively by screening the winning horses’ urine to make sure it is clean from these drugs.

## Experimental

### Instrumentation

HPLC Agilent 1260 Infinity Series Liquid Chromatography (Waldhorn, Germany). It consisted of a quaternary pump (model VL G7111A), a variable wavelength detector (VWD) (model G7114A), and a Zobrax Eclipse XDB—C18 column (5 µm, 150 × 4.6 mm). Jenway 3510, a pH meter (UK by Cole-Parmer) with a combination glass electrode, carried out pH measurements, and D-78224 Singen/ Htw.

### Samples

#### Pure samples

HBB and DIP were a generous gift from Arab Company for Medical products, Obour City, Egypt. Their purities were assessed to be 100.54 ± 1.30 and 100.33 ± 1.39, respectively, according to the reported method^38^. AMP was purchased from Loba Chemie, Mumbai, Maharashtra, India. TRO was supplied from Alfa Aesar, Germany. PHEN and DIC were purchased from Merck, Germany.

#### Market samples

Buscovet Compsitum injectable solution ®, labelled to contain 0.40 g/100 mL HBB and 50.00 g/100 mL DIP, was manufactured by the Arab Company for Medical Products in Egypt.

### Chemicals and reagents

- Methanol: HPLC grade (Merck, Germany)
- 0.02 M Phosphate buffer pH 5.4 ± 0.1: Prepared by dissolving 2.72 g of potassium dihydrogen phosphate (Adwic, Cairo, Egypt) in 800 mL distilled water. The pH was adjusted with 1N potassium hydroxide solution (Adwic, Cairo, Egypt) then it was diluted to 1000 mL with distilled water^14^.
- Two healthy 400 kg male Cows’ urine was collected. The cows were reared in an open system, with free access to food and water. They were provided by a local farm in Fayoum, Egypt.

### Standard solutions

#### Standard stock solutions of DIP, HBB, AMP, TRO, Phenol, and DIC as internal standard (2.00 mg/mL)

Standard stock solutions (2 mg/mL) of DIP, HBB, AMP, PHEN, DIC, and TRO were prepared by accurately weighing 200.00 mg of each into six separate 100- mL volumetric flasks, 70 mL methanol was added, sonicated, and then completed to the mark using the same solvent. While working solutions for each were prepared in 100-mL volumetric flasks by dilution using the mobile phase consisting of methanol: 0.02 M phosphate buffer pH 5.4 ± 0.1 (26:74, v/v) to obtain a final concentration of 100.00 µg/mL for assaying the cited compounds in pure form.

For the assay in urine, accurate different volumes of each of the stock standards were withdrawn and diluted using the mobile phase to reach the needed concentrations of DIP (300.00–1500.00 µg/mL), HBB, AMP (50.00–1200.00 µg/mL), and DIC as internal standard (400.00 µg/mL) to be spiked in urine.

All standard solutions were kept in amber-coloured bottles in the refrigerator when they were not used to prevent their photodegradation^43^ except for HBB and TRO, which are not affected by photodegradation^44^.

### Procedures

#### Chromatographic conditions

The proposed method was carried out at ambient temperature on ZOBRAX eclipse column—C18 (4.6 × 150 mm, 5 µm). The mobile phase consisted of methanol: phosphate buffer pH 5.4 ± 0.1 was kept at a ratio (26:74, v/v). The mobile phase was filtered using 0.45 mm membrane filters and degassed by a sonicator for 30 min before use. The flow rate was kept at 1.3 mL/min till min 11.2, then changed to 1.6 mL/min from min 11.2 till the end of the separation; UV detection was performed at 220.0 nm for all the studied components.

#### Construction of calibration curve.

Accurately measured aliquots of DIP and HBB were separately and accurately transferred from their standard working solutions (100.00 µg/mL) into sets of separate 10-mL volumetric flasks. The volume was completed to the mark using the stated mobile phase to prepare solutions with 1.00–40.00 µg/mL concentrations and 2.50–50.00 µg/mL for DIP and HBB, respectively. Triplicates for each solution concentration were injected into the HPLC apparatus under the previously mentioned chromatographic conditions. Linear calibration curves were constructed for each DIP and HBB, relating the area under the curve to their corresponding concentrations, and the regression equations were computed.

#### Separation of the cited drugs from their impurities

Aliquots of DIP, HBB, AMP, PHEN, and TRO were transferred from their standard working solutions 100 µg/mL, and appropriate dilution was made with the stated mobile phase to reach a concentration of 40.00 µg/mL DIP and AMP, 2.50 µg/mL HBB, 2.50 µg/mL PHEN, and 1.00 µg/mL TRO. The drugs were then injected into the HPLC apparatus. The procedure was then completed, as stated before.

#### Application to pharmaceutical formulation using the proposed HPLC method

An aliquot of Buscovet Compsitum injectable solution equivalent to 2.50 mL was accurately transferred using a micropipette into a 100 mL volumetric flask and completed to the mark using the stated mobile phase to obtain a stock mixture solution of 12.50 mg/mL DIP and 100.00 µg/mL HBB, then sonicated in an ultrasonic bath for 30 min. An appropriate dilution was made using the stated mobile phase to prepare a final concentration of 15.00 µg/mL of HBB for its determination, then 0.16 mL was accurately transferred into 100 mL volumetric flask from the stock solution and completed to the mark using the described mobile phase to a final concentration of 20.00 µg/mL of DIP for its assay. Then, the procedure was completed as described in the construction of calibration curves. Recoveries were calculated by dividing the practical concentration obtained from the regression equation by the theoretical concentration and multiplying it by 100.

$${\text{Recovery }}\left( \% \right) \, = {\text{ Actual Conc}}. \, \left( {{\text{practical Conc}}.} \right) \, /{\text{Theoretical Conc}}. \times { 1}00$$

#### Application of the proposed HPLC method for the determination of DIP, HBB, and AMP using DIC as an internal standard in spiked urine samples

A volume of 900.00 µL of free cow’s urine was transferred into test tubes, then 100.00 µL of different concentrations of working standard solutions of DIP (300.00–1500.00 µg/mL), HBB (50.00–1200.00 µg/mL), AMP (50.00–1200.00 µg/mL), and DIC as internal standard (400.00 µg/mL) were spiked, each solution was diluted with 1.00 mL of the mobile phase and filtered using syringe filter (pore size 0.45 micron) to reach concentrations of (15.00–75.00 µg/mL) DIP, (2.50–60.00 µg/mL) HBB and AMP and (20.00 µg/mL) DIC. Afterwards, the chromatographic procedures were followed, and the peak area of each drug was divided by the peak area of DIC as IS to get relative peak areas. Linear calibration curves were constructed for each DIP, HBB and AMP, relating the relative peak areas to their corresponding concentrations and the regression equations were computed.

#### Application of the proposed HPLC method for the determination of DIP, HBB and AMP using DIC as an internal standard *in real* urine samples

Two healthy 400 kg cows from a local Egyptian farm in El Fayoum reared in an opened system were each injected once with 20.00 mL of the pharmaceutical dosage form Buscovet Compsitum injectable solution ® by intramuscular route. Then, 200.00 mL urine samples from the midstream were collected by clean catch method using a specimen cup in the presence of a veterinarian after 3 h.

All experimental protocols and studies were approved by the research ethics committee for experimental and clinical studies at the Faculty of Pharmacy, Cairo University, and given the approval serial number AC (3339). All methods were carried out in accordance with relevant guidelines and regulations, and all the methods were reported in accordance with ARRIVE guidelines.

A volume of 900.00 µL of the collected urine sample was transferred into a test tube, then 100.00 µL of DIC as internal standard (400.00 µg/mL) was spiked and then diluted with 1.00 mL of the mobile phase and filtered using a syringe filter. Then, the chromatographic procedures were followed. The peak areas of DIP, HBB and AMP were integrated using DIC as IS by dividing each peak area of the studied drugs by the peak area of the IS to get their relative peak areas. The corresponding concentration was calculated from the corresponding regression equations. Recoveries were calculated by dividing the practical concentration obtained from the regression equation by the theoretical concentration and multiplying it by 100.

$${\text{Recovery }}\left( \% \right)\, = \,{\text{Actual Conc}}. \, \left( {{\text{practical Conc}}.} \right) \, /{\text{Theoretical Conc}}. \times {1}00.$$

### Validation

The method of separation of DIP and HBB from their impurities and preservatives was validated according to the International Conference on Harmonization (ICH) guidelines^45^ in terms of system suitability, limit of detection (LOD), limit of quantitation (LOQ), linearity, robustness, accuracy, specificity, and precision. Also, the method was extended to separate DIP and HBB in the presence of AMP as a metabolite of DIP using DIC as an internal standard in bovine urine and was validated according to the FDA guidelines^46^ The standard bioanalytical method validation recommendation concerns linearity, recovery, sensitivity, accuracy, precision, and stability (short-term, post-preparative, freeze-and-thaw, and long-term stability). The validation was described in detail in the supplementary file section S.1.1.

### Statistical analysis

Statistical comparisons were made between the results obtained from analysis of DIP and HBB pure and spiked samples and those obtained by the reported method, using Student’s t-test and F values showing no significant difference in accuracy and precision at p = 0.05.

## Results and discussion

**Fig. 1:**
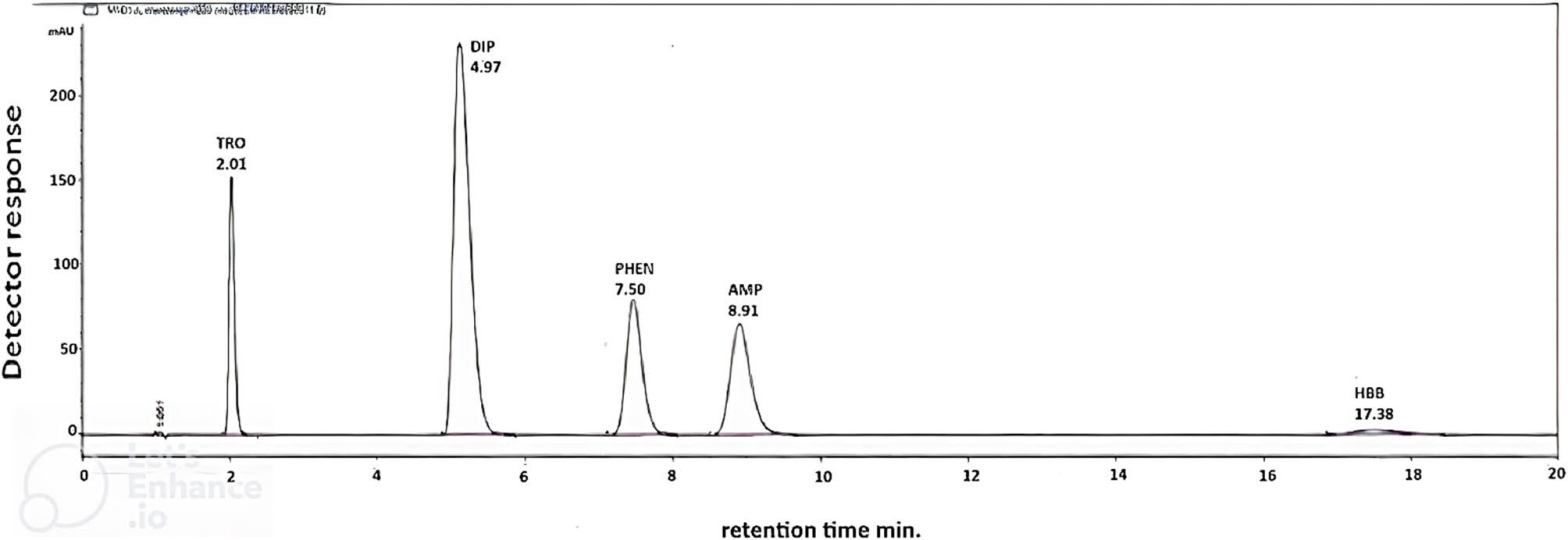
HPLC chromatogram showing [separation of 2 µg/mL DIP, 10 µg/mL HBB, 2 µg/mL AMP, 5 µg/mL Phenol and 1 µg/mL TRO with UV detection at 220 nm using methanol: phosphate buffer pH 5.4 ± 0.1 (26:74, v/v).

**Fig. 2:**
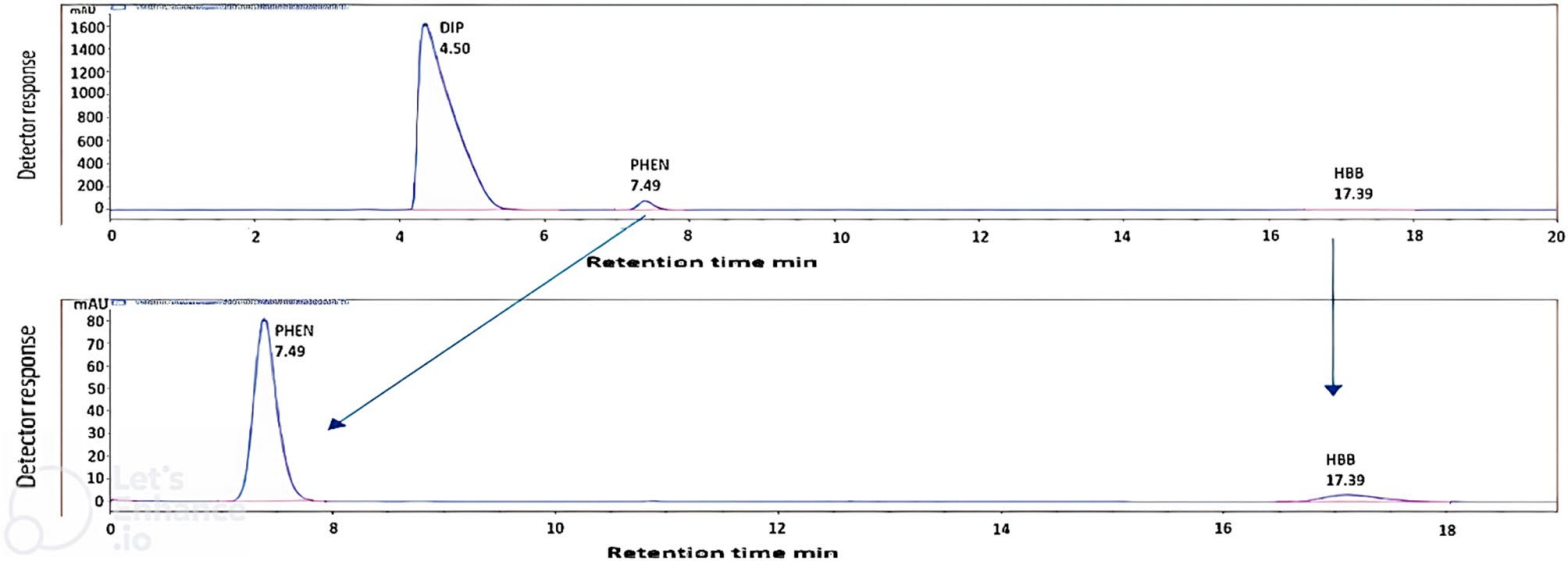
HPLC chromatogram of Buscovet compositum showing [HBB (10 µg/mL) and Phenol with UV detection at 220 nm using methanol: phosphate buffer pH 5.4 ± 0.1 (26:74, v/v).

After several trials, the simultaneous determination and separation of DIP and HBB in the presence of their officially related impurities was achieved. Different mobile phase compositions were tried using different organic solvents with different ratios, such as ethanol, methanol and acetonitrile. Different ways of elution and different column lengths (150 mm and 250 mm) were also studied, where symmetry and resolution were functions of judgment and reducing retention time. The optimum elution procedure was found to be an isocratic mobile phase consisting of methanol: phosphate buffer pH 5.4 ± 0.1 kept at a ratio (26:74, v/v). The pH of the phosphate buffer was of great importance as it critically affected the retention time and peak symmetry; pH 5.4 ± 0.1 was found to be suitable. Also, the usage of ZOBRAX eclipse XBD C18 column (4.6 × 150 mm, 5 µm) improves the peak shapes and resolution of DIP and HBB from AMP, TRO and PHEN at a suitable time, as shown in **(**Fig. 1**)** and for the separation of these components in the dosage form **(**Fig. 2**)**.

The mobile phase was introduced at a flow rate of 1.30 mL/min till min 11.20, then changed to 1.60 mL/min from min 11.20 till the end of the separation to decrease the run time without compromising separation and resolution, with UV detection at 220.00 nm. Under these optimum chromatographic conditions, separation of the cited compounds was achieved with retention times 2.01, 4.97, 7.50, 8.91 and 17.36 min for TRO, DIP, -PHEN, AMP and HBB, respectively.

To ensure maximum performance, system suitability parameters were determined by calculating capacity factor (k̀), resolution (Rs), selectivity (α) and the number of theoretical plates (N) for the HPLC method and the system was proved to be suitable relative to the reference values according to USP 35^6^ as illustrated in (Table S1). The method proved to be robust by analysis of the cited drugs under minor variations of different experimental conditions such as flow rate, pH and mobile phase composition, showing %RSD of the resolution 0.576 for DIP from TRO, 0.862 for DIP from PHEN and 0.525 for HBB from AMP. Also, the %RSD of capacity factor showed 1.144 for DIP and 0.540 for HBB, and for the RSD of the % assay, 0.784 for DIP and 1.204 for HBB^47,48^ (Table S2).

Validation of the proposed chromatographic method was done according to the ICH guidelines^45^, including linearity, accuracy, specificity and precision, as shown in (Table 1).

**Table 1:**
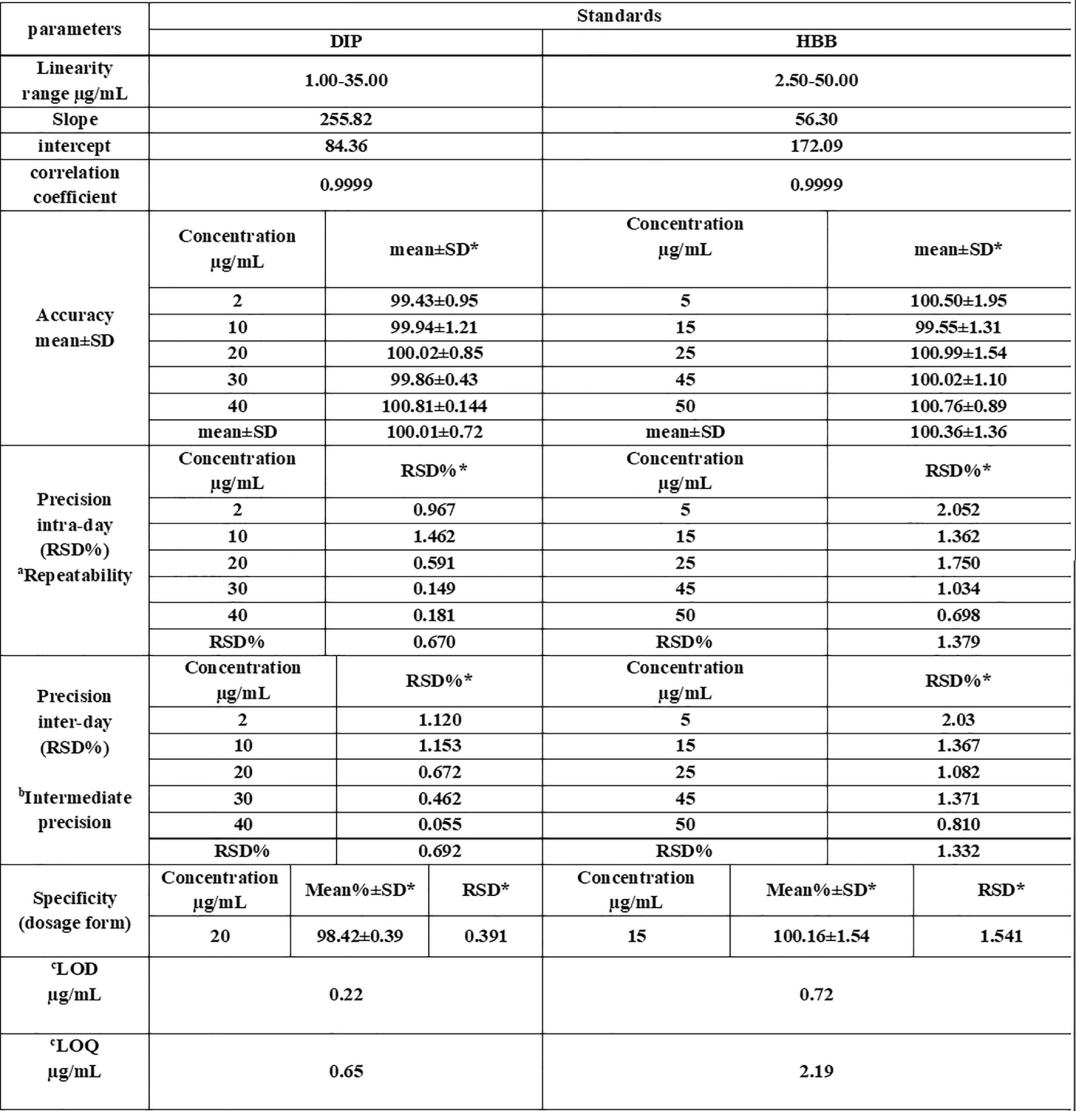
Regression and validation parameters of the proposed HPLC method for determining pure DIP and HBB samples.

*Average of three determinations

aThe intraday (n = 5) relative standard deviation of concentrations (2,10,20,30,35 ug/mL) of DIP and (5,15,25,45,50 ug/mL) of HBB repeated three times within the day.

bThe interday (n = 5) relative standard deviation of concentrations (2,10,20,30,35 ug/mL) of DIP and (5,15,25,45,50 ug/mL) of HBB repeated three times in three days.

ca Calculated from equation [LOD = 3.3 (SD / S), LOQ = 10 (SD / S); where SD is the standard deviation of intercept, and S is the slope of the calibration-curve.

The proposed method's accuracy was checked by applying it to five different concentrations of both DIP and HBB and then their corresponding concentrations were calculated from the regression equations. This proved the proposed method's high degree of accuracy over the linearity range.

The precision of the proposed method was determined by the analysis of five different concentrations of DIP and HBB in triplicates on a single-day intraday precision (repeatability) with RSD% 0.892 and 1.543 for DIP and HBB, respectively and on three consecutive days interday precision (intermediate precision) with RSD% 0.862 and 1.289 for DIP and HBB, respectively.

**Fig. 3:**
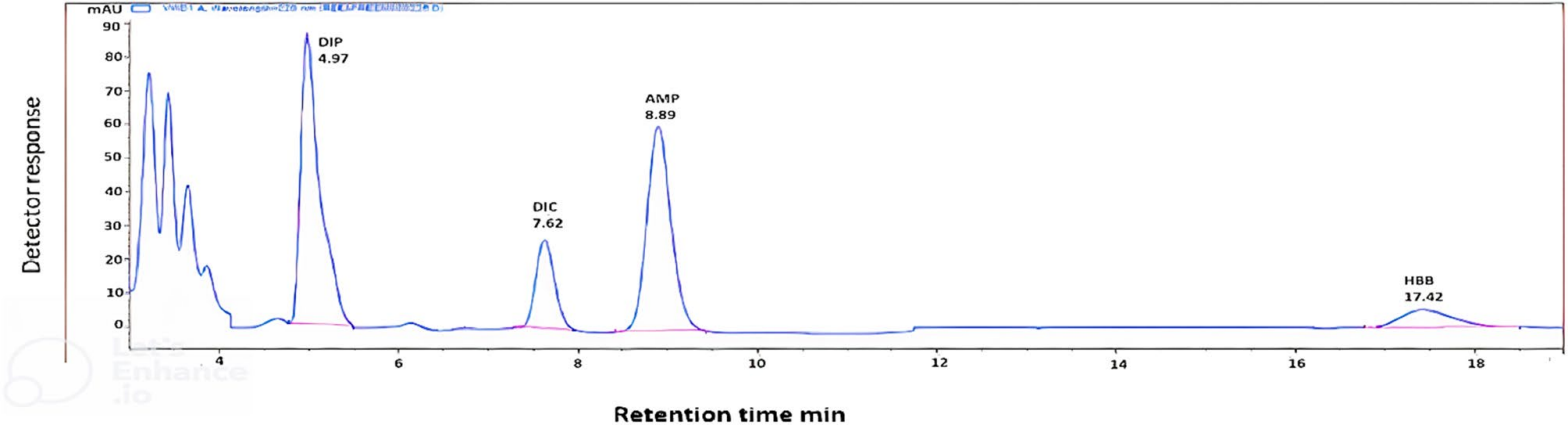
HPLC chromatogram of [DIP, DIC, AMP and HBB spiked in urine using methanol: phosphate buffer pH 5.4 ± 0.1 (26:74, v/v). UV detection at 220 nm is zoomed in, and urine noise is cropped.

The specificity of the suggested techniques was achieved by the proposed method's ability to determine the DIP and HBB in the presence of their commonly found impurities and the preservative (Fig. 2) and in their pharmaceutical preparation **(**Fig. 3**)** without interference with the excipients commonly present. Satisfactory resolution values support the specificity of the proposed method.

The method was applied for the analysis of the drugs mentioned above in dosage form, and results obtained by the suggested method were statistically compared to those obtained by the reported method^38^ for DIP and HBB, the calculated Student’s t-test and F values were less than the tabulated ones, demonstrating no significant difference concerning accuracy and precision at p = 0.05 **(**Table 2**).**

**Table 2:** Statistical comparison of the results obtained by the proposed method and the reported method for the determination of DIP and HBB in the dosage form.

Parameters	DIP		HBB	
	Proposed method	Reported method**^38^	Proposed method	Reported method**^38^
Mean***	98.42	99.31	100.16	99.92
SD	0.39	0.82	1.54	1.20
Variance	0.15	0.67	2.37	1.44
n	5	5	5	5
F value (6.39) *	4.48		1.65	
Student t-test (2.306) *	2.198		0.275	

*Values between parenthesis are the theoretical value of t and F at P = 0.05 and *n* = 5.

**HPLC method using C_18_ (250 mm × 4.6 mm) mobile phase composed of (water: methanol, 50:50) pH adjusted to 7 using triethylamine and trifluoracetic acid UV detection 210 nm ^38^.

***Percentage recoveries of 5 determinations.

Furthermore, the developed HPLC procedure was applied to assay DIP, HBB and AMP in bovine urine. DIP and HBB are used as spasmolytic drugs in cows. According to the Committee for Veterinary Medicinal Products (June 2003)^27^, after a single IV administration of DIP, concentrations of 4- methyl amino antipyrine, 4- amino antipyrine and 4- formyl amino antipyrine in urine samples taken after 4 h after treatment were found to be 1510.00, 90.00 and 16.00 µg/mL, respectively. It was also stated that after oral administration, no unchanged DIP is found in urine, but after IV and IM administration of DIP, a relatively slower process of DIP conversion and metabolism may allow a significant renal excretion of unchanged DIP^49^. On the other hand, upon surveying the metabolism and excretion of HBB, it was found that according to the Committee for Veterinary Medicinal Products (Nov. 1997)^18^. After the IV or IM administration of HBB, the concentration of unchanged HBB found in urine collected from cattle after 2–4 h. was 10.00 µg/mL.

**Fig. 4:**
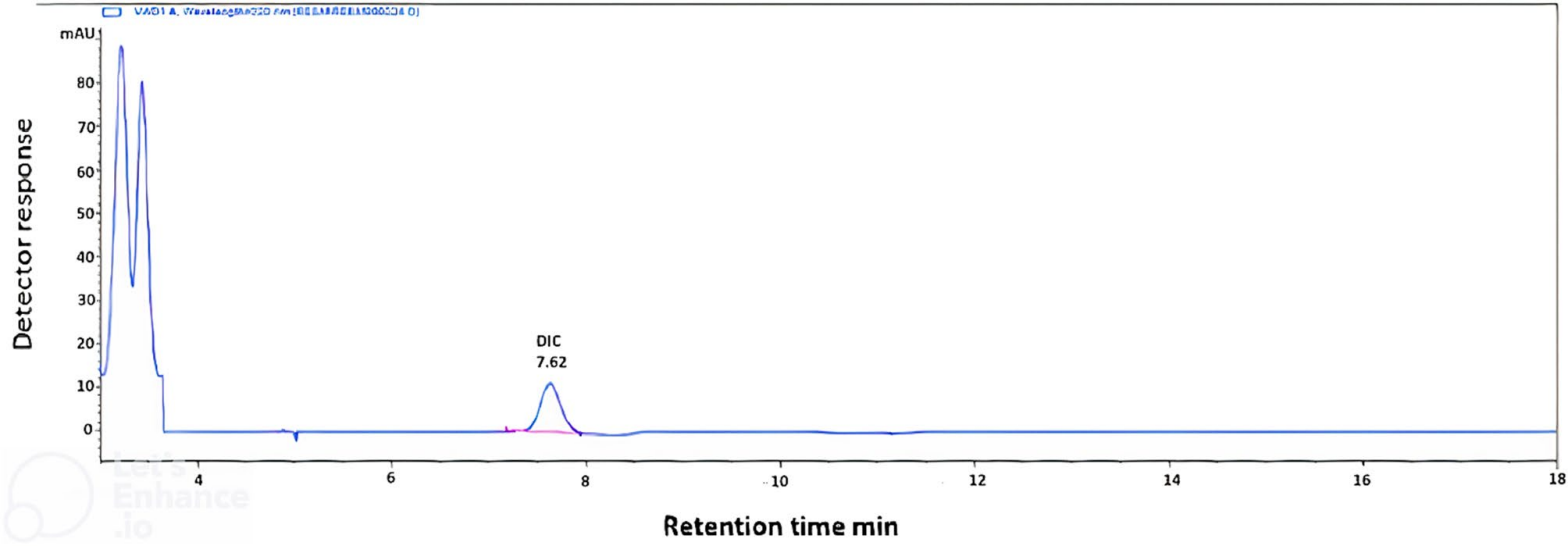
HPLC chromatogram of [DIC (20 µg/mL) spiked in urine blank using methanol: phosphate buffer pH 5.4 ± 0.1 (26:74, v/v). UV detection at 220 nm is zoomed in, and urine noise is cropped.

Under the stated optimized chromatographic conditions, good linear correlations between relative peak areas of DIP, HBB and AMP and their corresponding concentrations were successfully established in urine using DIC as an internal standard (having the same molar mass of DIP) and good separation was achieved **(**Fig. 3**)** without any interference from the blank urine samples spiked with DIC **(**Fig. 4**)** achieving low concentrations to detect the excretion of DIP (15.00–75.00 µg/mL), HBB (2.50–60.00 µg/mL) and AMP(2.50–60.00 µg/mL).

**Fig. 5:**
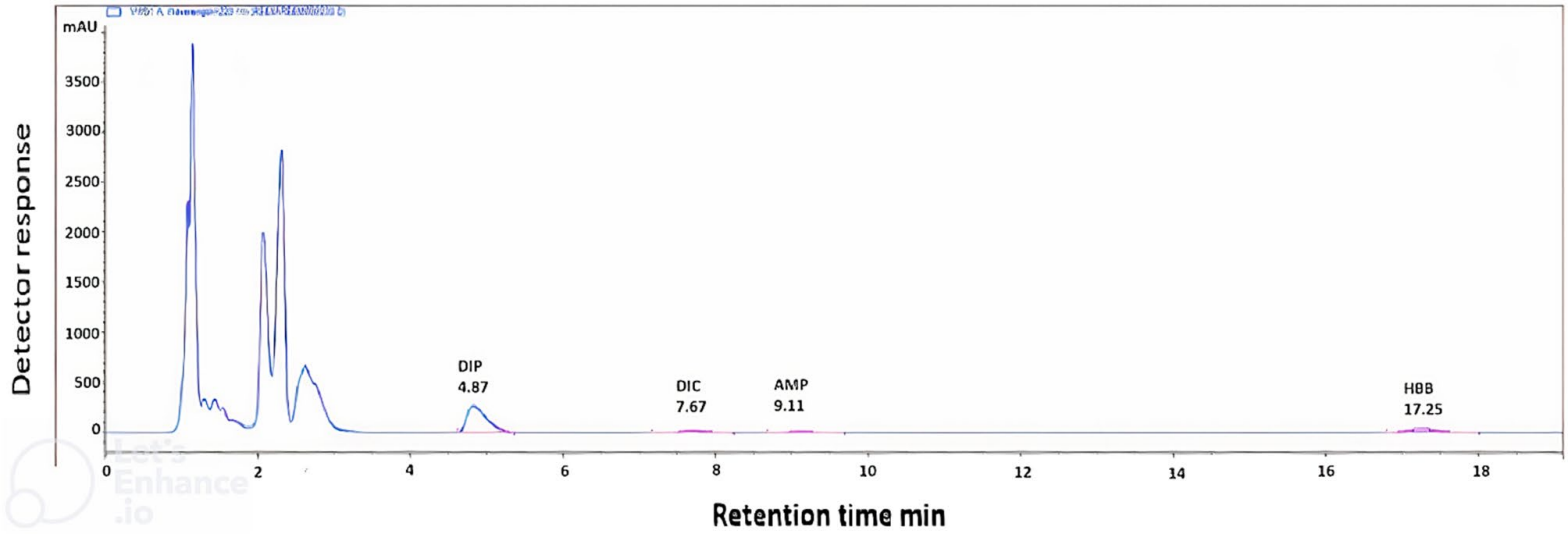
HPLC chromatogram of a real urine sample collected after 3 h. of treatment using methanol: phosphate buffer pH 5.4 ± 0.1 (26:74, v/v) UV detection at 220 nm.

Thus, the method ensured the detection of DIP (60.00 µg/mL), HBB (5.00 µg/mL) and AMP (30.00 µg/mL) in real urine samples collected 3 h after IM administration **(**Fig. 5**).**

Linearity and regression parameters of the proposed method of DIP, HBB and AMP spiking in urine and the validation of the method according to the bioanalytical validation stated by the ICH^50^ are stated in (Table 3). Accuracy and precision were calculated for LLOQ (lowest limit of quantitation), LQC, MQC and HQC (Low, Mid, and High-quality control); accuracy was found to be 100.30 ± 0.23 for DIP, 100.27 ± 0.41for HBB and 99.46 ± 0.48 for AMP. Three replicates of each of the concentrations of DIP, HBB and AMP were calculated on the same day for intraday precision with RSD% 0.332, 0.446, 0.426 for DIP, HBB and HBB, respectively and three separated days for interday precision with RSD% 0.124, 0.288 and 0.600 for DIP, HBB and HBB, respectively using the regression equations.

**Table 3:** Regression and validation parameters of the proposed HPLC method for the determination of pure DIP, HBB and AMP in bovine urine.

Parameters	Standards		
	DIP	HBB	AMP
Range µg/mL	15–75	2.5–60	2.5–60
Slope	1.1178	0.7914	0.1939
intercept	− 11.357	0.2956	0.2237
Correlation coefficient	0.9999	0.9998	0.9999
Accuracy ^a^mean ± SD	100.30 ± 0.23	100.27 ± 0.41	99.46 ± 0.48
Precision (RSD%) ^b^Repeatability ^c^Intermediate precision	0.332 0.124	0.446 0.288	0.426 0.600
^d^LLOQ µg/mL	15	2.5	2.5
^e^LQC µg/mL	45	7.5	7.5
^f^MQC µg/mL	55	25	25
^g^HQC µg/mL	60	50	50

^a^Mean recovery % and standard deviation of four determinations of DIP (15,45,55 and 60 ug/mL), HBB and AMP (2.5, 7.5, 25and 50 µg/mL).

^b^The intraday (*n* = 4) relative standard deviation of concentrations (15,45,55,60 ug/mL) of DIP and (2.5,7.5, 25,50 ug/mL) of HBB and AMP repeated three times within the day.

^c^The interday (*n* = 4) relative standard deviation of concentrations (15,45,55,60 ug/mL) of DIP and (2.5,7.5, 25,50 ug/mL) of HBB and AMP repeated three times in three days.

^d^Lowest limit of quantitation.

^e^Low-Quality Control.

^f^Middle-Quality Control.

^g^High-Quality Control.

To ensure maximum performance for the bioanalytical separation method, system suitability parameters were calculated and proved to be suitable relative to the reference values according to USP 35^6^ as illustrated in (Table S3).

It is of great importance for the bioanalytical method to quantitate and separate the analytes in the presence of other components that may be present in the matrix, including metabolites, impurities, degradation products, or matrix components. This can be done by analyzing six blank urine samples from different cows, and each sample is tested for any interference using the same chromatographic conditions **(**Fig. 4**)**. No interference from proteins or other metabolites was observed. Also, the separation of the four DIP, HBB, AMP and DIC peaks with satisfactory resolution was tested.

The extraction recoveries of DIP, HBB, and AMP from urine were examined by comparing the mean peak area of triplicates of four unextracted samples of drugs (LLOQ, LQC, MQC and HQC) to peak areas of the same concentrations with prepared spiked urine (14.00, 45.00, 55.00 and 60.00 µg/mL) for DIP and (2.50, 7.50, 25.00 and 50.00 µg/mL) for HBB and AMP **(**Table 4**)**.

**Table 4:** Recovery results of simultaneous determination of DIP, HBB and AMP in bovine urine by proposed method.

DIP		HBB		AMP	
Recovery%	RSD%	Recovery%	RSD%	Recovery%	RSD%
90.99	1.463	93.81	0.696	93.33	1.282
83.83	1.237	81.82	1.191	84.97	0.587
84.61	0.309	95.66	0.537	91.98	1.396
84.80	0.263	91.65	0.382	92.72	0.154

The extraction recoveries of DIP, HBB and AMP from urine were examined by comparing the mean peak area of triplicates of four unextracted samples of drugs (LLOQ, LQC, MQC and HQC) to peak areas of the same concentrations with prepared spiked urine (14.00, 45.00, 55.00 and 60.00 µg/mL) for DIP and (2.50, 7.50, 25.00 and 50.00 µg/mL) for HBB and AMP.

The stability of the drugs and the matrix, including storage conditions and chemical properties, were tested in four ways (short-term, long-term, post-preparative, freeze–thaw stability) by analyzing triplicates of concentrations of spiked drugs in urine (LLOQ, LQC, MQC and HQC) (14.00, 45.00, 55.00 and 60.00 µg/mL) for DIP and (2.50, 7.50, 25.00 and 50.00 µg/mL) for HBB and AMP and the method was found to be stable with satisfactory mean recoveries and RSD % (Table S4).

The results obtained for the analysis of spiked DIP (15.00, 45.00, 55.00 and 60.00 µg/mL) and HBB (2.50, 7.50, 25.00 and 50.00 µg/mL) in urine by the suggested method were statistically compared to those obtained by the reported method^51^ for DIP and HBB. The calculated Student’s t-test and F values were less than the tabulated ones, demonstrating no significant difference concerning accuracy and precision (Table S5).

**Fig. 6:**
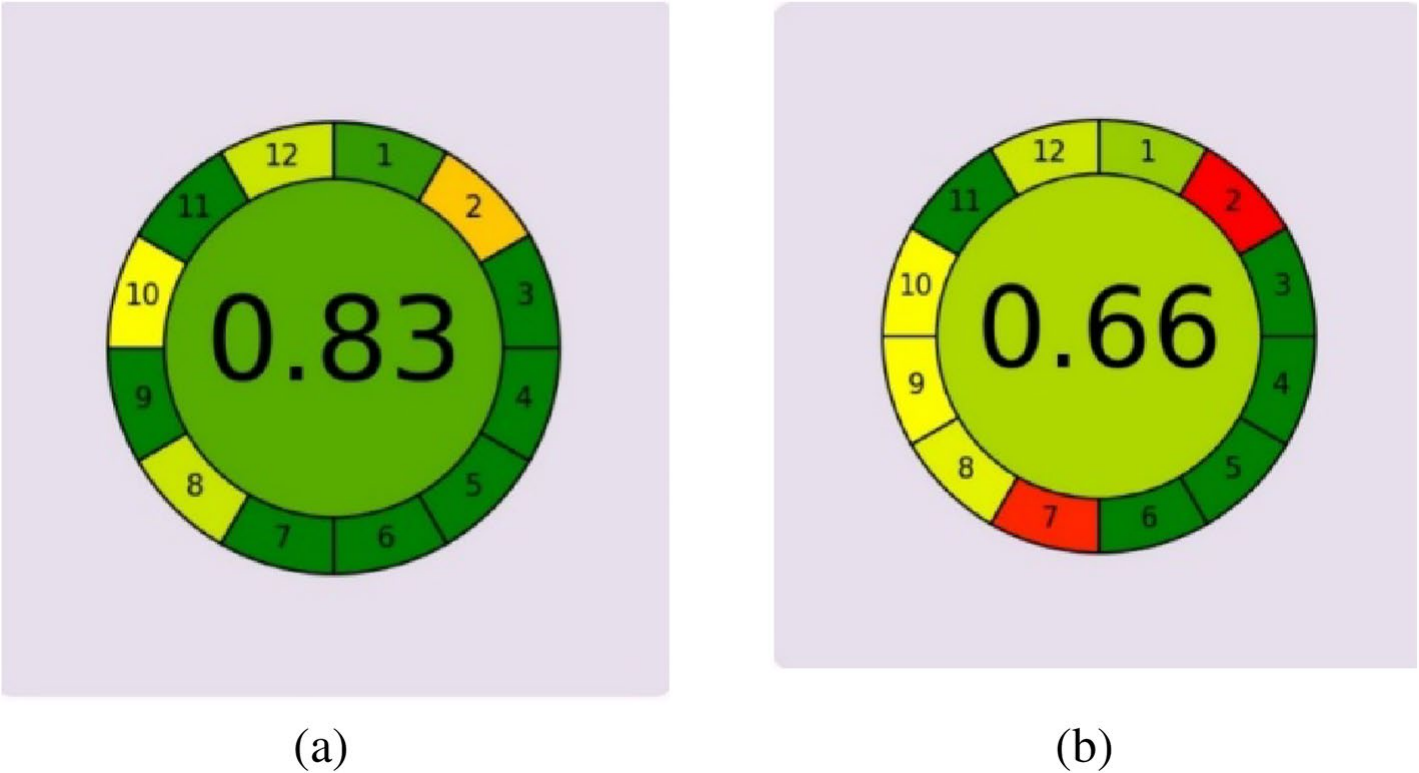
Score obtained for comparing the proposed HPLC method (**a**) to the reported method (**b**)^38^ using the AGREE calculator tool for the assessment of the greenness of the method.

The proposed method's Greenness has been assessed using two different tools (Analytical eco-scale and AGREE-Analytical greenness metric approach and software). The analytical eco scale (Table S6) depends on deducting penalty points depending on the parameters of an analytical method that disagrees with the ideal green method, which has a score of 100^52^. Upon calculating the penalty points of the proposed method, the score was found to be 83. AGREE calculator is an integral, flexible and straightforward assessment approach based on 12 principles of green analytical chemistry that are calculated and transformed into a 0–1 scale^53^, the suggested method scored 0.83 on the AGREE calculator and software (Fig. 6).

Upon comparing the suggested method to other reported methods that determine DIP and HBB using spectrophotometry^27^, TLC densitometry^36^ or HPLC^38^, the suggested approach appears to be superior. HPLC determination of DIP and HBB, particularly in the presence of their stated impurities, offers several green advantages over traditional methods like spectrophotometry or TLC regarding solvent usage, analysis time, automation, compatibility with green chemistry principles, and versatility. By adopting HPLC techniques, laboratories can significantly reduce their environmental footprint while maintaining high analytical performance and accuracy. Upon comparing the suggested method to the reported method^38^, the suggested method appeared to be greener and more efficient in terms of using less methanol in the mobile phase 26% in comparison to the 50%, which not only benefits the environment by conserving resources and minimizing pollution but also offers economic and regulatory advantages this was proved by comparing the two methods using AGREE software the suggested method score was 0.83 in comparison to 0.66 of the reported method **(**Fig. 6**)**. Embracing such greener practices aligns with broader sustainability goals and promotes responsible laboratory management. Also, the suggested method is the only reported method to separate the APIs in the presence of their impurities. It is crucial as regulatory agencies such as the FDA and EMA have strict guidelines regarding the allowable levels of impurities in pharmaceutical products. Hence, analyzing impurities is essential for compliance with these regulations. The method was further applied to determine DIP and HBB in the presence of DIP’s main active metabolite AMP in urine, which is essential in screening racehorses for doping or screening for drug abuse, which is considered the first reported method for this purpose.

## Conclusion

The suggested validated chromatographic method was proved to be selective, fast, accurate, precise, eco-friendly, and reproducible for the quantitative analysis and separation of DIP and HBB from their impurities (TRO, AMP) and Phenol as a preservative, which is essential in quality control labs and regulatory agencies as FDA and EMA to ensure that the pharmaceutical dosage form will maintain high quality and achieve the intended pharmacological effect with the least adverse effects. Moreover, it proved applicable for studying the excretion pattern and drug monitoring through the separation of DIP, HBB and AMP in bovine urine by an easy and efficient method and reaching excretion concentrations which the FEI can use in screening horses to detect doping for clean sport or to detect drug abuse in an easy and applicable way.

## Supplementary Information

Supplementary Information.

## Data Availability

The authors declare that the data supporting the findings of this study are available within the paper and its Supplementary Information files. Should any raw data files be needed in another format they are available from the corresponding author upon reasonable request.
