# Supplementary material for: “Eco-friendly HPLC method for analysis of dipyrone and hyoscine in different matrices with biomonitoring”
Source: Sci Rep. 2024 Sep 18;14:21775. doi: 10.1038/s41598-024-71046-6 (PMC11410794; doi:10.1038/s41598-024-71046-6)
Supplement: Supplementary file 1 — Supplementary Information. [file 41598_2024_71046_MOESM1_ESM.pdf]

"Eco-friendly HPLC method for analysis of dipyrone and hyoscine in different matrices with biomonitoring"

Reem A. el kalla [a], Nermine S. Ghoniem [b*], Hala E. Zaazaa [b], Ahmed Emad El Gendy [a], Ghada A. Sedik [b].

a Analytical Chemistry Department, Faculty of Pharmacy, Misr International University, Km 28 Misr-Ismailia Road, Cairo-Egypt

b Pharmaceutical Analytical Chemistry Department, Faculty of Pharmacy, Cairo University, Kasr El Aini, Cairo 11562, Egypt

Reem A. El Kalla: Reem.ibrahim@miuegypt.edu.eg
Dr. Nermine S. Ghoniem*: Nermine.ghoniem@pharma.cu.edu.eg
Prof. Dr. Hala E. Zaazaa: Hala.zaazaa@pharma.cu.edu.eg
Prof. Dr. Ahmed Emad El Gendy: Ahmed.emad@miuegypt.edu.eg
Assoc. Prof. Ghada A.Hamid Sedik: Ghada.sedik@pharma.cu.edu.eg

List of contents:

content		page
S.1.	Experimental………………………………………………………………...	1
S.1.1.	Validation…………………………………………………………………	1
S.1.2.	Bioanalytical method validation………………………………………	2
S.1.2.1.	Linearity and Range…………………………………………………....	2
S.1.2.2.	Lower limit of quantitation (LLOQ)…………………………………	2
S.1.2.3.	Selectivity……………………………………………………………...	2
S.1.2.4.	Accuracy and precision………………………………………………	3
S.1.2.5.	Extraction recovery……………………………………………………	3
S.1.2.6.	Stability…………………………………………………………………	3
S.2.	Results and Discussion………………………………………………	4
S.2.1	Validation…………………………………………………………………	4
S.2.2.	Bioanalytical method validation………………………………………	4
S.2.2.1.	Linearity and Range…………………………………………………....	4
S.2.2.2.	Lower limit of quantitation (LLOQ)…………………………………	4
S.2.2.3.	Selectivity…………………………………………………………………	5
S.2.2.4.	Accuracy and Recovery………………………………………………..	5
S.2.2.5.	Inter and intra-day precision………………………………………...	5
S.2.2.6.	Extraction recovery……………………………………………………	5
S.2.2.7.	Stability………………………………………………………………....	5

List of tables

Table S1: System suitability parameters of the developed HPLC method for the determination of DIP, HBB and their impurities.

Table S2: Robustness of the proposed HPLC method for determination of DIP and HBB and some impurities.

Table S3: System suitability parameters of the developed HPLC method for the determination of DIP, HBB and AMP in urine

Table S4: Stability results of simultaneous determination of DIP, HBB and AMP in bovine urine by the proposed method

Table S5: Statistical comparison of the results obtained by the proposed method and the reported method for the determination of DIP and HBB in urine

Table S6: Penalty points used in the Analytical eco-scale tool for the assessment of the greenness of the method

List of Figures

Fig. S1: Chemical structures of DIP (a), HBB (b), AMP (c), TRO (d) and DIC (e)

Supporting information

S.1. Experimental

S.1.1. Validation

The method validation was performed according to the ICH guidelines [1] for the analysis of the two drugs DIP and HBB in their pure form and their combined dosage form by preparing mixtures of the two drugs containing 1.00, 5.00, 15.00, 25.00, 35.00 and 40.00 µg/mL for DIP and 2.50, 10.00, 20.00, 30.00, 40.00 and 50.00 µg/mL for HBB each concentration was injected three times and chromatographed using the optimum condition, the calibration curve was plotted for each drug using the peak area versus the corresponding concentration. The limit of detection (LOD) and limit of quantification (LOQ) were calculated by equations (S1, S2):

$LOD = 3 \times S\ N$ (S1)

$LOQ = 10 \times S\ N$ (S2)

Where S/N is the signal-to-noise ratio

The method specificity is the ability to separate the three drugs with peaks of good resolution without any interference from the pharmaceutical preparation's excipients.

The inter-day and intra-day precisions were evaluated using a mixture of the two drugs using five concentrations (2,10,20,30,35 ug/mL) of DIP and (5,15,25,45,50 ug/mL) of HBB. Regarding the intra-day precision, each concentration was injected in triplicate on the same day, while in the inter-day precision, each concentration was injected on three consecutive days. The results were evaluated using percentage relative standard deviation (% RSD).

The accuracy of the method was determined by using a mixture of the two drugs of concentrations (2,10,20,30,35 ug/mL) of DIP and (5,15,25,45,50 ug/mL) of HBB and the results were calculated as %R. Accuracy (%) = Actual Conc. (practical Conc.)/Theoretical Conc. × 100

Method robustness is identified as the proposed method's ability to be unaffected by deliberate changes in the chromatographic conditions. The studied conditions were the pH value of the mobile phase, flow rate, and the percentage of aqueous: organic solvent in the mobile phase. The results were evaluated by the %RSD.

The method's system suitability parameters, including retention factor, resolution, selectivity factor, capacity factor, theoretical plate count, and the height equivalent to each theoretical plate, were tested.

S.1.2. Bioanalytical method validation

Bioanalytical validation of the suggested method was done according to the FDA guidelines [2] to ensure that the suggested method is satisfactory for its intended purpose, starting with the sample preparation step and continuing through the process.

S.1.2.1. Linearity and range

The method's linearity was investigated using nine non-zero samples of DIP, HBB, and AMP spiked in urine. In the range of (15-75 µg/mL) for DIP, (2.5-60 µg/mL) for HBB and (2.5-60 µg/mL) for AMP. In addition, six blank urine samples were analyzed to ensure the absence of any interferences.

The calibration curves were plotted using each analyte concentration against the corresponding relative peak area after dividing each drug's peak area by the peak area of the IS DIC to get relative peak areas. The acceptance criterion of the calibration curve is the regression coefficient (R^2) with a value of 0.99 or better, and each calculated standard concentration must be within 15.0 % deviation from the nominal value except for LLOQ, where the acceptable 100 deviation was 20.0 %.

S.1.2.2. Lower limit of quantification (LLOQ)

It is the lowest concentration in the calibration curve, with accuracy from 80.0 to 120.0 %. At the same time, its % RSD is less than 20.0 %.

S.1.2.3. Selectivity

It was evaluated by injecting six blank urine samples obtained from different cows to ensure that the analytes were not interfered with by the urine matrix.

S.1.2.4. Accuracy and precision

Accuracy was evaluated by % R of the QC samples, and the accepted range was as mentioned in section S.1.2.1. For intra-day precision, QC samples were analyzed six times on the same day, and each QC sample was analyzed six times on six different days for inter-day precision. The results were evaluated by % RSD, and the accepted range was as mentioned in section S.1.2.1.

S.1.2.5. Extraction recovery

The extraction recoveries of DIP, HBB, and AMP from urine were examined by comparing the mean peak area of triplicates of four unextracted samples of drugs (LLOQ, LQC, MQC and HQC) to peak areas of the same concentrations with prepared spiked urine (14.00, 45.00, 55.00 and 60.00 µg/mL) for DIP and (2.50, 7.50, 25.00 and 50.00 µg/mL) for HBB and AMP and % R was calculated as in equation (S3).

$$\textit{peak area of the analytes extracted from plasma samples} / \textit{peak area of the pure standard of the analytes} \times 100 \quad (S3)$$

S.1.2.6. Stability

QC samples were injected in triplicates for all conditions and then compared to freshly prepared samples.

Short term: LLOQ, LQC, MQC, and HQC samples were thawed at room temperature and kept at this temperature for 4-6 hours, then analyzed.

Post-preparative: The stability of the prepared samples was investigated by keeping three replicates of the LLOQ, LQC, MQC, and HQC samples at room temperature for 24 hours.

Long-term stability was examined by storing three aliquots of LLOQ, LQC, MQC, and HQC concentrations of the studied drugs at -20 °C for 30 days, then thawing them at room temperature and analyzing them.

Freeze and thaw: Three aliquots at each of LLOQ, LQC, MQC, and HQC samples were stored at -20°C for 24 hours and thawed at room temperature. When completely thawed, the samples were refrozen for 24 hours under the same conditions. The freeze and thaw cycles were repeated three

times and then analyzed. The results were evaluated by % deviation from the freshly prepared samples using equation (S4), where the deviation should be ± 15 %.

$\% R\ of\ stored\ sample/\% R\ of\ the\ freshly\ prepared\ sample\ \% R\ of\ the\ freshly\ prepared\ sample \times 100$ (S4)

S.2. Result and Discussion

S.2.1. Validation

The method was linear in the range (1.00-35.00 µg/mL) for DIP and (2.50-50.00 µg/mL) for HBB and exhibited a satisfactory linearity regression R (0.9999) for both drugs with LOD 0.22 µg/mL and 0.72 µg/mL and LOQ 0.65 µg/mL and 2.19 µg/mL for DIP and HBB, respectively, Table1. Also, the method was selective due to its ability to separate the two drugs in the presence of their impurities and preservatives without any interference.

The results in Table S2 showed that the method was robust due to its ability to resist small changes in the chromatographic conditions. Finally, the method passes the system suitability parameters in Table S1.

S.2.2. Bioanalytical validation

S.2.2.1. Linearity and range

The linearity range was found to be (15-75 µg/mL) for DIP, (2.5-60 µg/mL) for HBB, and (2.5-60 µg/mL) for AMP with linearity regression R (0.9999-0.9998).

S.2.2.2. Lower limit of quantification (LLOQ)

The LLOQ was 15.00, 2.50, and 2.50 µg/mL for DIP, HBB, and AMP. Accuracy was 100.30±0.23 for DIP, 100.27±0.41for HBB, and 99.46±0.48 for AMP. Three replicates of each of the concentrations of DIP, HBB, and AMP were calculated on the same day for intraday precision with RSD% 0.332, 0.446, 0.426 for DIP, HBB, and HBB, respectively, and three separated days for interday precision with RSD% 0.124, 0.288 and 0.600 for DIP, HBB and HBB, respectively using the regression equations.

S.2.3.3. Selectivity

The method was selective, as the blank urine samples were chromatographed without interferences. In addition, it is possible to separate the two main drugs along with DIP metabolite AMP and DIC as IS with sharp peaks without any interferents from the urine proteins, as shown in (Fig.4).

S.2.3.4. Accuracy and precision

The method was accurate and precise, as shown in Table 3.

S.2.3.5. Extraction recovery

The extraction protocol of the three drugs from the urine samples showed high efficiency, as represented in Table 4.

S.2.3.6. Stability

Samples showed good stability, as shown in Table S4.

Supplementary tables

Table S1: System suitability parameters of the developed HPLC method for the determination of DIP, HBB and their impurities

parameters	drugs					References [3]
	TRO	DIP	PHEN	AMP	HBB	
**Selectivity (α)**	4.03 TRO		1.64 PHEN	2.07 AMP		α>1.00
**Resolution (Rs)**	18.57 TRO		10.81 PHEN	19.93 AMP		R>2.00
**Symmetry factor**	1.01	1.02	0.99	0.98	1.03	0.80-1.20
**Number of theoretical plates**	6464	97171	22500	15035	15620	Increase with efficiency of separation
**HETP (cm/plate)**	0.0023	0.0001	0.0007	0.0010	0.0010	The smaller the value, the higher the efficiency

Table S2: Robustness of the proposed HPLC method for determination of DIP and HBB and their impurities

Robustness parameters		R_s			K		% assay	
		DIP		HBB From AMP	DIP	HBB	DIP	HBB
		From TRO	From PHEN					
**Flow rate**	**1.3 +0.1 mL/min**	18.54	10.75	19.80	3.77	15.60	101.20	98.47
	**1.3 - 0.1 mL/min**	18.65	10.90	20.01	3.85	15.80	98.99	101.45
**pH value**	**5.4 +0.1 unit**	18.55	10.76	19.95	3.80	15.78	100.44	100.99
	**5.4 - 0.1 unit**	18.65	10.85	19.95	3.82	15.75	99.98	99.60
**Mobile Phase Methanol : Phosphate Buffer (26:74)**	**27:73**	18.51	10.67	19.87	3.86	15.67	100.90	100.77
	**25:75**	18.80	10.90	20.10	3.75	15.82	99.99	98.96
**%RSD**		0.576	0.862	0.525	1.144	0.540	0.784	1.204

Table S3: System suitability parameters of the developed HPLC method for the determination of DIP, HBB and AMP in urine

parameters	HPLC				References [3]
	DIP	HBB	AMP		
**Selectivity (α)**	1.67	2.09	1.19		α>1
**Resolution (Rs)**	11.30 DICLO	18.12 AMP	18.12 HBB	4.72 DICLO	R>2
**Symmetry factor**	0.93	0.92	1.10		0.8-1.2
**Number of theoretical plates**	12102	11966	13350		Increase with efficiency of separation
**HETP (cm/plate)**	0.0012	0.0013	0.0011		The smaller the value, the higher the efficiency

Table S4: Stability results of simultaneous determination of DIP, HBB and AMP in bovine urine by the proposed method

stability	DIP		HBB		AMP	
	Mean Recovery%*	Mean RSD%**	Mean Recovery%*	Mean RSD%**	Mean Recovery%*	Mean RSD%**
[a] Short term	100.31	0.260	101.26	0.875	100.63	0.712
[b] Post preparative	100.23	0.593	100.54	0.718	100.32	0.522
[c] Long term	100.63	0.785	99.73	0.501	100.05	0.687
[d] Freeze-thaw (Three cycles)	100.51	0.638	99.73	0.501	100.18	0.727

[a] LLOQ, LQC, MQC, and HQC samples were thawed at room temperature and kept at this temperature for 4-6 hours, then analyzed

[b] The stability of the prepared samples was investigated by keeping three replicates of the LLOQ, LQC, MQC, and HQC samples at room temperature for 24 hours.

[c] Long-term stability was examined by storing three aliquots of LLOQ, LQC, MQC, and HQC concentrations of the studied drugs at -20 °C for 30 days. Then, they were thawed at room temperature and analyzed.

[d] three aliquots at each of LLOQ, LQC, MQC, and HQC samples were stored at -20°C for 24 hours and thawed at room temperature. When completely thawed, the samples were refrozen for 24 hours under the same conditions. The freeze and thaw cycles were repeated three times and then analyzed.

The concentrations used for measuring the stability of DIP (15,45,55 and 60 ug/mL), HBB, and AMP (2.5, 7.5, 25and 50 µg/mL)

Table S5: Statistical comparison of the results obtained by the proposed method and the reported method [4] for the determination of DIP and HBB in urine

Parameters	DIP		HBB	
	Proposed method	Reported method**	Proposed method	Reported method**
**Mean**	100.30	100.54	100.27	100.33
**SD**	1.35	1.81	1.38	1.52
**Variance**	1.82	3.28	1.90	2.31
**n**	5	5	5	5
**F value (6.39) ***	1.80		1.22	
**Student t-test (2.306) ***	0.237		0.065	

* Values between parenthesis are the theoretical value of t and F at P= 0.05 and n= 5

** HPLC method using C_{18} (250 mm x 4.6 mm) mobile phase composed of (water: methanol, 50:50) pH adjusted to 7 using triethylamine and trifluoroacetic acid Uv detection 210 nm.

Table S6: Penalty points used in the Analytical eco-scale tool for the assessment of the greenness of the method.

Parameters	Penalty points
**Yield**	**(100-effective yield)/2**
**Price of the reaction components (to obtain 10 mmol)**	
Inexpensive (< 10 USD)	0
Expensive (between 10 and 50 USD)	3
Very expensive (>50 USD)	5
**Safety (adapted for the globally harmonized system of classification and labelling of chemicals)**	
GHS09 (dangerous for the environment)	5
GHS06 (toxic)	5
GHS02 (flammable)	5
GHS01 (explosive)	10
GHS07, GHS08 (extremely toxic)	10
**Technical setup**	
Common setup	0
Instruments for controlled addition (dropping funnel, etc.)	1
Unconventional activation technique (microwave, etc.)	2
Pressure equipment > 1 atm	3
Any additional special glassware	1
(inert) gas atmosphere	1
Glove box	3
**Temperature/ Time**	
Room temperature <1hr	0
Room temperature <24hr	1
Heating < 1hr	2
Heating > 1hr	3
Cooling to 0 °C	4
Cooling to < 0°C	5
**Workup/ purification**	
None	0
Cooling to room temperature	0
Adding solvent	0
Simple filtration	0
Removing of solvent with b.p. <150°C	0
Crystallization and filtration	1
Removing of solvent with b.p. >150°C	2
Solid phase extraction	2
Distillation	3
Sublimation	3
Liquid-liquid extraction	3
Classical chromatography	10

Supplementary figures

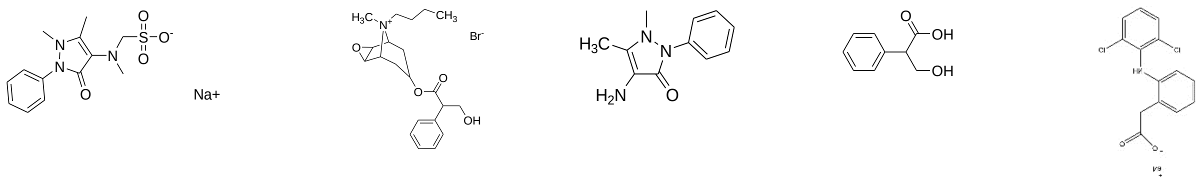

(a) (b) (c) (d) (e)

Fig. S1: Chemical structures of DIP (a), HBB (b), AMP (c), TRO (d) and DIC (e) [3].

References

1. Branch, S.K., *Guidelines from the international conference on harmonisation (ICH). Journal of pharmaceutical and biomedical analysis*, (2005). **38**(5): p. 798-805.

2. Swartz, M. and I. Krull, *Validation of bioanalytical methods—highlights of FDA's guidance. LCGC North America*, (2003). **21**(2): p. 136.

3. Pharmacopea,U.S. and N.Formulary. *USP 35-NF 30*. 2011. United States Pharmacopeial Convention Inc., Rockville Maryland, USA.

4. Gamal, M., *Simultaneous Determination Of Hyoscine Butyl Bromide and Dipyrone in Their Binary Mixture By RP-HPLC Method. Journal of Pharmacy and Biological Sciences*, (2012). **3**(5): p. 29-36.
